# Digital healthcare in COPD management: a narrative review on the advantages, pitfalls, and need for further research

**DOI:** 10.1177/17534666221075493

**Published:** 2022-03-02

**Authors:** Alastair Watson, Tom M.A. Wilkinson

**Affiliations:** Clinical and Experimental Sciences, Faculty of Medicine, University of Southampton, Southampton, UKNIHR Southampton Biomedical Research Centre, University Hospital Southampton, Southampton, UKCollege of Medical and Dental Sciences, University of Birmingham, Birmingham, UK; Clinical and Experimental Sciences, Faculty of Medicine, University of Southampton, Southampton SO16 6YD, UK. NIHR Southampton Biomedical Research Centre, University Hospital Southampton, Southampton, UK

**Keywords:** COPD, digital health, exacerbations, inhaler technique, machine learning, patient engagement, pulmonary rehabilitation

## Abstract

Chronic obstructive pulmonary disease (COPD) remains a leading cause of morbidity and mortality despite current treatment strategies which focus on smoking cessation, pulmonary rehabilitation, and symptomatic relief. A focus of COPD care is to encourage self-management, particularly during COVID-19, where much face-to-face care has been reduced or ceased. Digital health solutions may offer affordable and scalable solutions to support COPD patient education and self-management, such solutions could improve clinical outcomes and expand service reach for limited additional cost. However, optimal ways to deliver digital medicine are still in development, and there are a number of important considerations for clinicians, commissioners, and patients to ensure successful implementation of digitally augmented care. In this narrative review, we discuss advantages, pitfalls, and future prospects of digital healthcare, which offer a variety of tools including self-management plans, education videos, inhaler training videos, feedback to patients and healthcare professionals (HCPs), exacerbation monitoring, and pulmonary rehabilitation. We discuss the key issues with sustaining patient and HCP engagement and limiting attrition of use, interoperability with devices, integration into healthcare systems, and ensuring inclusivity and accessibility. We explore the essential areas of research beyond determining safety and efficacy to understand the acceptability of digital healthcare solutions to patients, clinicians, and healthcare systems, and hence ways to improve this and sustain engagement. Finally, we explore the regulatory challenges to ensure quality and engagement and effective integration into current healthcare systems and care pathways, while maintaining patients’ autonomy and privacy. Understanding and addressing these issues and successful incorporation of an acceptable, simple, scalable, affordable, and future-proof digital solution into healthcare systems could help remodel global chronic disease management and fractured healthcare systems to provide best patient care and optimisation of healthcare resources to meet the global burden and unmet clinical need of COPD.

## Introduction: COPD remains a great under-recognised and unmet clinical need

Chronic obstructive pulmonary disease (COPD) is still a leading cause of morbidity and mortality worldwide, despite current treatment strategies and the international evidenced-based guidelines for care.^1–5^ In spite of prescribed therapy, the overall outcomes from COPD remain poor with acute exacerbations of COPD (AECOPD) being a major driver of morbidity, mortality, and disease progression.^6–11^ The economic costs of this are significant, and the European Union estimated direct costs to account for 3% (€38.6 billion) of their total healthcare budget, wider societal costs are likely to be much greater.^12^ Current treatment pathways to try and mitigate this burden focus on early diagnosis and risk factor prevention (including smoking cessation), symptom management, pulmonary rehabilitation (PR),^13^ maintaining a healthy weight, routine vaccinations,^14–17^ oxygen therapy for certain patients,^18–20^ and identification and management of AECOPD.^21,22^ Early recognition of exacerbation symptoms is essential,^23–29^ and interventions to enable prompt detection of exacerbations and underlying patient-specific drivers of exacerbations to prevent readmissions are desperately needed.^30–34^

### Barriers to COPD care

Due to the chronic nature of COPD, education, skills provision, and encouragement for patients to self-manage their condition has become a key part of COPD care.^35^ Patients with COPD have a number of required tasks to effectively self-manage their condition, including recognising and responding to symptoms (self-actualization), which improves patients’ early recognition and treatment of AECOPD.^36^ Patients are also required to recognise and avoid exacerbation triggers and understand and adhere to treatment plans.

Complexities in delivering this approach to COPD management at the patient level contribute to suboptimal outcomes including poor adherence to inhaled medications, inadequate inhaler technique, limited disease understanding, and limited skills to self-manage; poor access to services also carries adverse impacts. It is estimated that only 40–60% of patients adhere to their prescribed regime, and 10% of patients with a metered dose inhaler perform all essential steps to take it correctly, highlighting the essential role of education and inhaler technique training.^37^ Patients with COPD are often required to adapt work and lifestyle around their symptoms, maintain adequate exercise and diet, refrain from smoking, and look after self-wellbeing and mental health.^37^ To highlight the issues that patients with COPD face with the accessibility to healthcare services to support self-management, a British Lung foundation report suggests patients are not receiving the right support for these roles and various perceived barriers have been reported.^12^ PR is also an important aspect of COPD treatment. However, problems with access to and engagement with PR are widely reported, the 2019 UK National COPD Audit Programme found that only 54% of patients with stable COPD started PR within 90 days of referral and 33% of patients being referred did not attend an initial PR assessment in 2017.^14,15^ Multifaceted, engaging, scalable, and affordable approaches to allow accessibility to PR, education and self-management plans to support behavioural changes are needed to overcome these problems and enable effective patient self-management.^16^

To support patients with COPD, healthcare providers (HCPs) also have various essential roles which are time-consuming and resource intensive, particularly when reliant on paper-based records and face-to-face visits.^17^ These include: recognition and reviewing of patients’ symptoms, clinical information and AECOPD; tailoring and engaging patients to optimal treatment plans; providing preventive strategies and services; and inspiring patients to modify lifestyle.^17^ With an ageing world population and resultant increased pressure on health service resources, which has been exacerbated by the pandemic, innovative solutions need to be found.^18–20^

### Challenges to COPD management in low- and middle-income countries

Particular problems in delivering COPD management are faced in low- and middle-income countries (LMICs), where COPD burden is highest due to the exposure to open fires or stoves fuelled by kerosene, coal, or biomass from very young ages.^5,21–23^ LMICs often have under-resourced healthcare systems which are poorly equipped to deliver optimal respiratory care with a lack of access to: diagnostic tools including spirometry; respiratory care specialists, resulting in most care being delivered by primary care nursing staff; preventive measures including vaccinations, nutritional support and smoking cessation services; longitudinal surveillance data; research around outcomes; and public health and educational policies.^23^ In this context, scalable, affordable, and inclusive solutions are required to address this need.

## Aims

In the peri-COVID-19 era, where much of the face-to-face services have been reduced or even ceased, there is an even greater need for health service solutions which are accessible 24/7 and can empower patients and provide them with the required skills to effectively manage their COPD in the future.^24–26^ Digital healthcare is an emerging area of medicine which may have potential in meeting this unmet clinical need. These rapidly evolving innovations have a wide variety of new applications, as demonstrated by a range of small feasibility studies which have recently been published. In this narrative review, we, therefore, aimed to highlight some of these advancements and provide some interesting discussion points around the different approaches which digital health innovations can take, without undertaking formal appraisal of each study. Furthermore, we aimed to highlight some of the pitfalls to digital healthcare innovations, the barriers to engagement and global adoption of effective and safe innovations. Finally, we explore the essential areas of research now required for the facilitation of future systematic reviews and high-quality evidence, to facilitate regulation and adoption of digital innovations for digitally augmented COPD care in the future.

## Methods

We performed a narrative review using independent search strategies, developed by AW and checked by TW, to identify relavent studies which demonstrated the potential of digital health innovations for use as self-management plans, providing PR, improving inhaler technique, and identifying exacerbations to allow early treatment (Table S1). The results for these search strategies are given in Tables S2, S3, S4, and S5. We also performed a literature search to identify the potential of digital health in COVID-19 (search strategy given in Table S1). Additional articles were identified through reading relavent reviews and reference lists from studies found through our formal search strategy. In addition to this formal search strategy, we performed a wide general literature search around digital health in COPD, with an aim of broadly reviewing and generating new insights around the potential advantages, pitfalls, and barriers for utilisation of digital health innovations.

Relavent studies for inclusion within the narrative of this review were identified by AW and TW, any conflicts of opinions about which studies should be included were resolved through discussion. As this was a narrative review, aimed at generating insights and discussing key findings and attributes of studies from a broad literature base, we did not formally assess study quality using, for example, the RoB 2 tool.^27^ Furthermore, no formal protocol was written or published for this narrative review, for example, on The International Prospective Register of Systematic Reviews (PROSPERO),^28^ and the Preferred Reporting Items for Systematic Reviews and Meta-Analyses (PRISMA) guidelines for systematic review reporting were not used.^29^

## Digital health applications, a potential opportunity for remodelling COPD care

### Introduction to digital health applications

**Figure 1.**
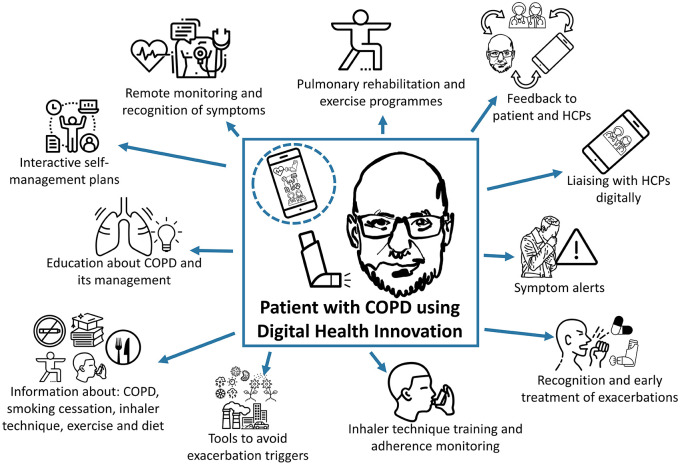
Potential opportunities for digital innovations to facilitate effective COPD management. Figure was formed from images taken from The Noun Project. The Noun Project, 8800 Venice Blvd., Los Angeles, CA 90034. Work is licenced under the Attribution 3.0 Unported (CC BY 3.0). Images used were downloaded from https://thenounproject.com on 26th and 27th of July 2021, and include (in the central box); Man by Ariel Kotzer in the handrawn faces Collection, used two times within figure; Inhaler by Line Icons Pro, Hyderabad; Mobile by LAFS, RU, used three times within figure; (within the mobile) Nurses by Bold Yellow, US, used three times within figure; Management by Massupa Kaewgahya, TH in the Leadership and Management Collection; Inhaler by Jino, CN, used two times within figure; (from top left clockwise) Management by Massupa Kaewgahya, TH in the Leadership and Management Collection; Diagnosis by Nhor; Heart rate by Symbolon, IT; Yoga by Becris, used two times in the figure; Arrows by Atif Arshad, AE, used three times; Cough by Hey Rabbit, TH in the Virus Transmission Collection; Alert by Emma Mitchell; Cough by Ari Sandi, ID; Antibiotics by UNiCORN; Asthma by Parkjisun in the Diseases Outline Collection, used two times in the figure; Virus by Koson Rattanaphan, TH in the Vaccine and Laboratory Collection; Bacteria by Helen Wong, UK in the Single cells organisms Collection; Pollen by Vectors Point, PK in the Set of Nature and Outdoor Line Vector Icons Collection; Pollution by Chameleon Design, IN in the Country 1 Collection; Pollution by David Carapinha, PT in the Ecology Collection; Weather by Sumit Saengthong, TH in the Weather Collection; Food by Guilherme Furtado, BR; NoSmoking by Vectors Market; Education by Wichai Wi, TH; Light Bulb by Deemak Daksina, ID; Lungs by Andrei Yushencko.

Effective, safe, accessible, and engaging digital healthcare solutions which are able to be integrated into global healthcare systems may play a role in helping to meet this demand in COPD care needs. Digital interventions are unrestricted by individual practices or healthcare systems and come in a range of forms, including: synchronous applications (apps) which provide real-time video conferencing or telephone calls; asynchronous solutions such as emails, smartphone messages, or notifications; remote monitoring or recording devices, such as traditional telehealth interventions; information providing devices; and modern multi-tooled digital health apps which can facilitate behavioural changes and self-management. Digital health apps have the potential to provide a range of solutions, including: patient education programmes to support inhaler technique and modification of lifestyle factors; interactive self-management plans; systems to remote monitor; tools to record and communicate symptoms; and integration of environmental and physiological data to help understand an individual’s disease and modify risk factors, management and care accordingly (Figure 1). These are discussed in detail below.

### Digital COPD self-management plans

Self-management plans should be tailored to a patient’s needs, concerns, disease severity, and associated comorbidities and are known to be beneficial in COPD management.^30^ These can equip patients with the tools required to utilise resources effectively, problem solve, make decisions, form therapeutic relationships with HCPs, and recognise symptoms.^31,32^ Digital health apps have potential in complementing or replacing paper-based self-management plans in an accessible and scalable way in various settings, including primary care, outpatients, and inpatients. They can pose many advantages, including the ability to contain customisable and engaging content to facilitate self-management, to be able to capture and collate patient data and to be accessed remotely by clinicians.^33^ An early study looked at the effectiveness of a 3 month web-based self-management system comprised of online education, bespoke widgets (symptom alerts), and exercise and inhaler technique videos in 36 patients with COPD. This demonstrated a significant improvement in a validated COPD Assessment Test (CAT) questionnaire to measure COPD impact scores, and in inhaler technique.^34^ Since these promising early results, demonstrating proof of principle, a comprehensive digital care management tool and aid for HCPs called ‘myCOPD’ has been developed and is now endorsed by The National Institute for Health and Care Excellence (NICE).^35^ This tool provides not only a self-management plan but also educational videos to teach correct inhaler technique; tools to help patients understand when to take medications; a tool to cross-check prescribed medications; a COPD assessment tool to allow tracking and better control of symptoms; a pollution-level prediction tool and an online 6-week PR course. A randomised controlled trial (RCT) in the United Kingdom in 60 recently diagnosed patients with mild–moderate COPD demonstrated that in those receiving ‘myCOPD’ *vs* usual care there was an associated CAT score improvement with greater use of the digital self-management app.^36^ Furthermore, a RCT which, recruited patients with COPD after hospitalisation due to severe AECOPD, demonstrated a clinically meaningful lower CAT score after the 90-day study period .^40^

Other alternate platforms with potential for providing digital self-management plans have been developed. Farmer *et al.* undertook an RCT where the intervention arm was recommended to use the ‘EDGE’ digital health tablet system and to complete an adaptive symptom diary and measure their oxygen saturations by wireless pulse oximeter.^41,42^ This was as compared with those receiving usual care of a paper-based self-management plan. The EDGE platform allowed custom algorithms to be developed with personalised alert thresholds, and the RCT demonstrated good engagement with the platform being utilised six times a week on average. The RCT demonstrated a decrease in their primary outcome of Saint George’s Respiratory Questionnaire (SGRQ-C), with an estimated difference of −1.7 in the digital intervention arm. However, this was below the clinically meaningful decrease of 4 and not statistically significant. This RCT did, however, see an improvement in generic health status, as determined by an improvement in the EuroQol 5-Dimension Questionnaire (EQ-5D) score in the digital intervention arm of 0.076 (*p* = 0.03), as well as a reduction in the median number of visits to general practitioners and practice nurses for the digital intervention arm *versus* usual care, 4 *versus* 5.5 (*p* = 0.06) and 1.5 *versus* 2.5 (*p* = 0.03), respectively. However, statistically significant differences were not seen in other secondary outcomes. Despite results from these feasibility trials, issues around the internal validity and generalisability of these studies require consideration and are discussed throughout this article. For example, issues around the impact of selection bias in RCTs, which may include more self-motivated populations, are relavent throughout the studies within this article and requires some thought, as these populations may not be representative of the overall COPD population. Furthermore, larger cross-comparable RCTs to complement these initial results are required to confirm these improvements in clinical outcomes and to understand whether digital health apps can equip specific groups of patients with the knowledge and confidence for effective and safe self-management.

#### The potential of traditional telehealth for COPD self-management

Conventional telehealth interventions may also have potential in allowing patient monitoring and clinical decisions to be made. A survey of 65 responding COPD services from 52 different NHS trusts found that a third of services were using some form of telehealth intervention, including frequently monitoring oxygen saturation, heart rate, and breathlessness.^43^ Telehealth interventions can allow alarms to be triggered to notify clinicians during periods of symptom worsening. However, respondents believed that >40% of alarms were false positives.^43^ A systematic review reported that telehealth was beneficial in reducing hospitalisation and emergency department visits but mortality was actually greater than usual care.^44^ A further review reported conflicting outcomes from studies, some reporting improved patient outcomes with telemonitoring and others reporting no effect.^45^ A large 12-month RCT (*n* = 3320), using a second-generation telehealth intervention, demonstrated no beneficial effect in improving psychological outcomes, including health-related quality of life, anxiety, or depressive symptoms for patients with COPD, diabetes, or heart failure.^46^ Other companies like Medvivo are offering integrated digital health and care services, including general practitioner (GP) out-of-hour services and telehealth and other technology-enabled care solutions. These have been brought into increasing focus during COVID-19. A feasibility RCT, aimed at investigating the potential of Docobo HealthHUB home telemonitors to reduce health care utilisation *versus* standard of care, has also been undertaken in 40 stable, optimised patients with moderate to severe COPD. This study found that those receiving the intervention had fewer contacts with primary care for chest problems (*p* < 0.03), but there was no difference in emergency department visits.^47^ Traditional telehealth approaches may have potential in aiding COPD management. However, it is clear that greater standardisation is required to allow for more comparable high-quality evidence. Moreover, it may be that more modern, rapidly evolving, flexible, digital innovations which have been designed in a more patient-centred way with customisable tools could have a greater impact in engaging and empowering patients to facilitate behavioural changes and effective self-management, rather than simple traditional telehealth monitoring symptoms.^33^

#### A need for further evidence and a user-centred approach

Digital self-management interventions have also shown promise in asthma (*n* = 88) and a feasibility RCT recruited 88 patients and gave them either the digital health intervention ‘My Breathing Matters’ or usual care and demonstrated similar improvements in asthma-related quality-of-life score.^48^ Further qualitative evaluation of this intervention confirmed patients’ perceived benefits to using this intervention and identified factors influencing user engagement and areas for future improvement, including providing a rationale to patients for tools and bonus features which are unlockable dependent on the patient’s wishes.^49^ Using a complementary qualitative approach for COPD digital self-management plans and a user-centred and iterative approach to the design of digital health interventions will likely be important to enhance effective engagement, we further discuss this later in the engagement section of this review.^50,51^

### Potential to improve inhaler use in COPD

Digital healthcare apps offer a range of utilities which could potentially explain the positive trial outcomes. Monitoring and education of patients’ inhaler techniques are critical components of COPD self-management.^52^ Smart inhalers and inhaler add-ons have now been developed to measure not only adherence but also to assess technique. These could be useful in monitoring and rectifying unintentional poor adherence, particularly when coupled with home training videos.^53,54^ A 120-day study gave patients inhaler monitoring software which sent dose reminders and provided usage feedback. This demonstrated significant improvements in inhaler technique, alongside clinically relevant improvements in CAT scores, SGRQ and 6-minute walk test (6MWT). Furthermore, they saw adherence rates at 94.3%, well above the 10–40% reported average.^55,56^ However, this was a single-armed trial with an undisclosed number of patients in a published abstract, and this remains to be demonstrated in a larger RCT.^55^

The ‘myCOPD’ app provides educational videos on inhaler technique and the RCT by North *et al.*,^39^ which recruited patients following AECOPD, demonstrated fewer critical errors after the 90-day study period. Similarly, Crooks *et al.*,^36^ demonstrated lower odds of having ⩾1 critical inhaler error after 90 days in patients recently diagnosed with mild-moderate COPD. Although these RCTs report adherence to the digital health apps and CAT score improvements in association with digital app use, these and most other studies do not report the impact of a digital health app on adherence to their inhaler or self-management regime, nor the engagement of patients specifically with inhaler technique training videos. Incorporation of these endpoints in future clinical trial design using reliable quantitative methods is essential to provide the evidence base to determine relative contributions of each aspect of digital health solutions for optimal future design of digital tools for COPD care.^57^

The utility of smart devices and Bluetooth inhalers to monitor inhaler use and technique may also provide useful information and guide COPD management in different care settings.^58^ A 12-month one-armed feasibility study enrolled 201 patients with COPD and used the ‘Propeller’ inhaler sensor to track date, time, and frequency of Short-Acting Beta Agonists (SABA) use, as well as providing disease-management information.^59^ This study demonstrated a 49% decrease in SABA use after 6 months, with an increase in symptom-free days.^59^ A further study demonstrated increased inhaler use following AECOPD.^60^ However, interventional RCTs using these technologies to discern whether they lead to sustained changes in self-management and patient outcomes are lacking.^61^ Furthermore, a greater understanding around how this information can be linked to other patient data, such as patient records, to offer personalised treatment plans is required.

Integration of inhaler adherence and inhaler technique data could provide essential feedback to patients to help them understand and self-manage their disease and appreciate how adherence to treatment regimens could improve their COPD symptoms. Engagement of patients through digital healthcare apps and provision of essential self-management skills could also increase patients’ feelings of self-control and impact experience and COPD management. As a result, digital healthcare apps could play a role in helping decrease chronic illness-related anxiety and depression, which have been shown to be closely linked with deterioration of COPD symptom experiences; these themselves have been shown to impact patient’s confidence, adherence, and ability to self-manage.^62,63^ However, the feasibility study by North *et al.*^40^ demonstrated similar Hospital Anxiety and Depression Questionnaire scores (HAD)^64^ in patients receiving the digital health app intervention *versus* standard management plans. Therefore, delineating whether intensive monitoring using digital healthcare apps is beneficial is required, or whether it could have a negative effect and increase chronic disease-related anxiety or depression, or lead to undue additional self-management load which is unacceptable to patients.^65^ Beyond improving COPD adherence and management, smart inhaler devices and digital health apps could play an important role in decreasing healthcare resource utilisation. A preliminary study of 20 patients with COPD with increased healthcare utilisation demonstrated a decrease in annualised all-cause healthcare utilisation at 6 months.^66^ The broader long-term impact of multi-tooled digital healthcare devices on healthcare resource utilisation now needs to be determined, as well as the potential negative impacts on patients and HCPs.

### Supporting recognition and response to COPD exacerbations

Early recognition of AECOPD is an essential part of COPD management and can limit their already significant burden on healthcare resources.^67,68^ Self-management plans are key to reducing the risk of AECOPD hospitalisations.^31,32^ Here, digital health apps could play a role in facilitating self-actualisation and improving COPD outcomes, by providing educational videos and self-management plans, as well as integrating symptom or physiological data. Many apps provide these tools, as well as a pollution forecast tool which integrates met office and DEFRA weather and pollution reports, to allow patients to respond to risks and manage their daily activities and COPD accordingly, to try to reduce AECOPD.^40,69,70^

Digital health apps could help to reduce numbers of AECOPD or increase the reaction of patients to them. The RESCUE study demonstrated a greater, albeit non-significant, reduction in exacerbation frequency in the digital health app arm with an incidence rate ratio of 0.58 for AECOPD and odds ratio of 0.383 for readmission rate. Another RCT investigated the use of an automated Internet-linked, monitoring and self-management support tablet system and demonstrated an overall improvement in generic health status, a non-significant decrease in median visits to GPs, and slight non-significant decrease in hospital admission over 12 months.^42,71^ Despite the potentially promising signals from these feasibility trials, the potential of these innovations to improve AECOPD outcomes through earlier recognition and intervention needs to now be fully delineated in larger highly powered longitudinal RCTs.

#### The potential of digital physiological sensors for helping to identify AECOPD

Pairing digital health apps with wearable physiological sensors to provide new detailed information about COPD symptoms and patient and provider feedback could be useful in identifying AECOPD and guiding management to improve patient quality of life.^72,73^ An RCT comparing the utility of the EDGE digital health system for improving COPD outcomes *versus* usual care of a paper-based self-management plan demonstrated that data on pulse rate, oxygen saturation, and respiratory rate, provided by use of a pulse oximeter, were predictive of exacerbations, with an area under the curve of 0.682.^74^ The monitoring and use of overnight pulse oximetry data has also shown potential in a proof-of-concept study where the study identified an increase in S_p_O_2_ entropy and decrease in long-term fractal-like exponent (α2) during an exacerbation *versus* stable disease phase. Receiver operating characteristic (ROC) analyses of these data demonstrated a capacity to classify COPD phases into either a stable or exacerbation phase with the best positive predictor value being 70% and negative predictor value of 78%. The potential of overnight pulse oximetry needs further exploration in larger studies. However, integration of such approaches into digital innovations may hold promise in the future.^75^

Other examples of sensors include accelerometers which are small, portable, functionally diverse, and accurate.^76–78^ A meta-analysis of 38 studies demonstrated that accelerometer-determined steps/day correlated with FEV1, and one study in 21 patients with COPD demonstrated an inverse correlation with hyperinflation.^79,80^ Moy *et al.*^81^ measured activity levels using an ankle-worn accelerometer to demonstrate an incremental increased risk of AECOPD and COPD hospitalisation with decreased activity. Studies have also demonstrated the use of accelerometer data in predicting readmissions following AECOPD discharge, and even risk of mortality.^82–84^ These studies demonstrate the utility of remote data monitoring and their potential in risk-stratifying patients. Integration of data from smart inhalers, physiological monitors, and other third-party sources such as weather and pollution could, not only provide essential information to guide self-management and clinicians’ practice, but allow digital biomarker identification to risk-stratify patients and prevent severe exacerbations.^33,85^

#### Artificial intelligence and machine learning approaches for predicting exacerbations

Intelligent analysis of personalised data has demonstrated utility and various algorithms, artificial intelligence (AI) and machine learning approaches have shown potential in, not only diagnosing COPD and identifying COPD endotypes, but predicting AECOPDs, readmissions, and outcomes using telemonitoring, health records, and imaging data.^74,86–93^ Orchard *et al.*^91^ utilised data from a large RCT to successfully predict COPD hospital admissions based on telemonitoring data with an area under the curve (AUC) of 0.74. Wang *et al.* compared five machine learning algorithms to predict AECOPD using electronic medical records and found a ROC curve of 0.90. Wu *et al.*^92^ undertook a cohort study to continually monitor 67 patients’ real-time lifestyle and indoor environment data from air quality-sensing devices, a smartphone app, and wearable devices and utilised supervised prediction algorithms to obtain an ROC curve of >0.9 in predicting AECOPD. This is a rapidly evolving and important area of research which, when paired with digital health innovations, could help revolutionise current COPD care for the better. However, much of this work has been undertaken in single-source populations without external validation, and some of these data sets have missing data which can lead to incorrect structures of models and biases in conclusions. Future consideration is required around: the ideal experimental design; ability to replicate and validate study findings; model interpretability; and how to categorise patients, define exacerbations, and select which model to use.^74,94^ Furthermore, the impact of implementation of these tools on burden of work for patients and clinicians is an important consideration and, alongside problems around integrating these new approaches into modern healthcare structures, is discussed below. Large prospective longitudinal studies and real-world data sets are required to optimise and validate these approaches and generate the evidence base to facilitate regulation, which is costly and may take some time. The generalisability of algorithms for different COPD severities and endotypes also needs to be demonstrated.

Machine learning approaches may also raise ethical issues around inequality and violations of privacy. Data sets which have been used thus far are based on populations from high-income countries, with very little research data being available and research being undertaken in populations in LMIC who have under-resourced healthcare systems and the highest need. These tools also require extensive training and computational resource which may limit their applicability. The lack of efficacy of some previous telehealth trials has limited the large implementations of this technology and machine learning approaches. Further work in this area is now essential to design optimised, validated, safe, and effective regulated AI technologies, able to be integrated into healthcare systems.

### Potential for accessible digital PR programmes

Physical inactivity is an independent risk factor for AECOPD and mortality.^79–81,83^ PR is an essential part of COPD management and can improve exercise capacity, health-related quality-of-life, and decrease healthcare resource utilisation.^95^ However, there are widely reported problems with suitability of programmes, due to accessibility impacting on both uptake and attrition; less than half of patients are reported to complete PR.^96–98^ Therefore, finding cost-effective ways of delivering engaging PR programmes to the wide COPD population are essential.

An RCT by Bourne *et al.*^58^ provided a 6-week incremental exercise programme and education sessions (*n* = 64) *versus* conventional face-to-face PR (*n* = 26) and demonstrated non-inferiority in 6MWT and CAT scores. Adherence was incomplete, however, accessed PR sessions were overall slightly greater than the face-to-face arm. Holland *et al.* demonstrated non-inferiority and were not able to rule out superiority in improvements in 6MWD, quality of life, and dyspnoea in 80 patients with COPD receiving home-based PR via telephone vs 86 patients receiving traditional centre-based PR at the end of the programme. Furthermore, these programmes had completion rates of 91% and 49%, respectively. Other studies used ‘Kaia COPD’, a digitalized PR smartphone delivery programme, and found a PR completion rate of 61% in 56 eligible users who downloaded the app and an improvement in CAT scores, but not in dyspnoea.^99,100^ An 8-week RCT in 154 subjects with moderate to severe COPD used an intervention of six times a week video-guided exercises, with weekly health coaching and monitoring by computer tablet through a platform by MinnHealth. This RCT found good adherence with 86% undertaking the proposed six times a week exercise routine. This study found no significant improvements in breathlessness in the intervention arm, their primary outcome, but saw significant improvements in self-management abilities (*p* < 0.001).^101^ These levels of completion compare favourably to usual care; the 2015 UK COPD PR audit reports that only 69% of patients referred for face-to-face PR attended an initial assessment and only 42% of patients completed the PR programme, thus highlighting the need to find new ways to increase patient accessibility and engagement with PR such as digital health innovations.^98^ An RCT in asthma patients, which delivered a DVD and booklet-based physiotherapy breathing retraining intervention (*n* = 261), has also reported an improvement in Asthma Quality of Life Questionnaire scores compared with usual care (*n* = 262).^102^ However, these are small feasibility studies and engagement in the real-world setting may be significantly lower.

#### Potential limitations of digital PR and gaps in the evidence base

These preliminary studies suggest that PR and other physical treatment sessions may be safe and effective with delivery through home digital technologies and large longitudinal RCTs are now required. However, the majority of evidence, thus far, comes from small feasibility non-inferiority trials and the primary outcomes often do not meet recognised minimal clinically important changes, which limit comparisons with the gold standard of face-to-face PR. Furthermore, there is a lack of published evidence delineating the factors leading to decreased engagement with PR and attrition. Young *et al.*^103^ identified social isolation and active smoking to associate with non-adherence and that interactive group PR sessions could be effective. Further thought is now required to understand how best to improve engagement and integrate digital PR into current healthcare systems. Lewis *et al.* undertook a mixed methods service evaluation in patients suitable for PR with chronic respiratory diseases. In this evaluation, they found that 17/25 (68%) patients were able to transfer using a digital eLearn Moodle platform for provision of remote PR during COVID-19 and 14/25 (56%) completed PR.^51^ Furthermore, this study found improvements in various health-related questionaries, including primary Health Questionnaire-9 (CI: −0.3 to −5.1 (*p* = 0.029) and Chronic Respiratory Questionnaire dyspnoea (CI: 0.5–1.3 (*p* = 0.001)).^51^ The qualitative research within this evaluation demonstrated that patients perceived the online programme to be beneficial and to elicit functional improvements in activities of daily living. However, this study did highlight some of the key barriers to digital provision of PR, including: the lack of group interaction on the online platform and reluctance of participants to engage with each other without the presence of staff; the burden to staff and the need for their investment of time to ensure beneficial outcomes; the need for further innovation for the provided Moodle PR platform; the concern of staff around ensuring patient safety and exercise progression as a group; and the need for leadership to successfully provide digital PR.^51^ Considerations of training, engagement, and adaptation of staff to allow provision of digital PR warrants some consideration, these factors may be barriers to the use of digital PR in our current care models and need to be explored. Problems around inclusivity and accessibility are also important to consider and are discussed at length below, as well as issues around patient and staff digital literacy. The generalisability and internal validity of these findings are also important to consider, due to the potential for selection bias of motivated patients in RCTs within respiratory research, difficulty in blinding patients for digital interventions, and the impact of attrition and loss to follow-up on outcomes. Future studies should ensure complete outcome reporting, including baseline characteristics and outcomes of drop-outs; use intention-to-treat analysis; and should be undertaken in different COPD groups longitudinally in real-world clinical settings.

### The benefit of digital health apps in the COVID-19 era

The COVID-19 pandemic has caused unprecedented disruption to the delivery of health services and to daily life.^24,26,104–106^ With the requirement to mitigate the impact of COVID-19 on patients; shortage of personal protective equipment; and the reduction or cancellation of health services during COVID-19 peaks, digital health apps, which hold the potential to empower patients and provide them with the skills to facilitate self-management, have driven new interest from both care-givers and patients. Organisations have now started to rapidly scale up, synergise and expand digital health innovations.^107^ For example, a recent study reported the use of rapid optimisation methods to introduce a digital intervention aimed to change infection control behaviours during the COVID-19 pandemic.^108,109^ With health services now becoming re-established, the requirement for traditional face-to-face sessions as a standard are being re-examined. A recent study found that 66% of 335 survey respondents would consider changing their future face-to-face consultation to a telephone review.^110^ Thus many patients may prefer remote consultations, particularly when living in remote locations, here digital health apps have the potential to supplement these telephone consultations by providing unique insights into the patient’s COPD symptoms, adherence to medication and self-management behaviours.^111,112^ Now, with lengthened waiting lists and continued reductions in accessibility to healthcare services, digital healthcare innovations have the potential to increase accessibility to healthcare as well as improve the experience of patients and health professional.^113^ Digital health innovations could help triage patients requiring urgent consultations and postpone those which do not, and facilitate effective COPD care with a minimised risk of COVID-19 infection in patients with COPD who are at increased risk of poor COVID-19 outcomes; this may become particularly important with the potential emergence of new variants of concern.^107,114–116^

## The pitfalls of digital health innovations

### Introduction to the challenges of digital health innovations

Digital health innovations may help to provide health and social care in the future for an ageing population with increased healthcare needs. However, there are various challenges to be overcome, including ensuring: accessibility and inclusivity; digital literacy and skills; sustained patient and clinician engagement; scalability and successful integration into healthcare systems; building of the necessary evidence base; and effective regulation. These have been highlighted in the ‘NASSS framework which helps practitioners and commissioners to understand and address these challenges and identifies key challenges as being: digital health innovations **n**ot being adopted, or soon **a**bandoned by patients/healthcare professionals (HCPs); demonstration of utility at small scale and problems with **s**cale-up; **s**pread to other similar healthcare settings; and problems with **s**ustained use over time.^117^ In this section, we discuss these challenges and potential ways to overcome them to ensure the effective use of digital health innovations for COPD management.

### The challenge of ensuring accessibility and inclusivity

Digital solutions are unrestricted by individual practices or healthcare systems and have potential to be accessed by hard-to-reach populations globally, particularly in northern Europe where Internet use is at 96%. However, only 51% of the world population has access to Internet and only 24% in East Africa.^118,119^ There is therefore a real potential inclusivity problem around digital healthcare solutions, particularly due to the COPD burden in LMIC.^5,21–23^ Those without Internet access are more likely to be older and of lower socioeconomic backgrounds, common features of populations where COPD is more prevalent, and there is the most unmet clinical need.^23,120–123^ Internet availability and mobile phone availability is likely to increase over time. However, problems are faced with device costs, particularly in low socioeconomic and elderly populations.^124^ Innovations requiring specialised devices, compared with downloadable mobile apps, are therefore unlikely to be as inclusive, broadly scalable and easy to be incorporated into healthcare systems. More advanced technologies such as telehealthcare systems may be useful for specific populations but could further increase disparity in health resource availability.^125^ Digital literacy, visual and cognitive impairments, learning difficulties, limitations with manual dexterity and a lack of confidence are additional obstacles in older adults using, reading, and interpreting information from digital healthcare apps, these merit important consideration.^126^

Engagement with digital health apps is underrepresented in certain ethnic groups and older adults, and there is therefore a real risk of a digital divide, with tiered access to healthcare resources and resultant increase in health disparity in the United Kingdom and globally.^127^ Concerns have been raised about the potential for digital exclusion to worsen social exclusion; this has previously been discussed in detail.^128^ Importantly, social exclusion associates with negative COPD outcomes and is already a problem in elderly and underprivileged populations.^129,130^ However, digital innovations may provide an additional benefit in mitigating social exclusion through the introduction of Internet use to disadvantaged groups.^128^ Digital health apps have been proposed to help build social connectedness and virtual environments and promote positive health behaviours. However, access to and use of digital health innovations is often lowest for individuals who would benefit the most.^131^ Lessons need to be learnt about how to best meet the skills and resource gaps globally, as well as other barriers which need to be overcome.

#### A need for solutions to ensure accessibility and inclusivity

Intuitive voice interfaces may be beneficial to facilitate the engagement of elderly populations with digital health innovations through overcoming usability fears, a lack of confidence, and problems with vision and manual dexterity. Voice interfaces may further be able to serve a diagnostic role in the future through feedback of vocal biomarkers of neurological or mental health status changes to HCPs.^126,132^ Further understanding the complexities of ageing could allow integration of other innovative design features to facilitate the use of digital health apps in this population.^126^ Thought around inclusion of different languages may also lead to greater accessibility in LMIC, alongside people within the United Kingdom who do not have English as their first language; these populations already have unequal healthcare service access and disproportionate health outcomes.^133–135^

The COVID-19 pandemic has increased the use of digital technologies and dependence on digital services, leading digital spaces to develop from an amenity to a necessity.^136^ However, this has led to an increased dependence and stress on available services, including public digital resources and speed of Internet.^131,136^ Furthermore, the economic impact of COVID-19 may affect the ability of underprivileged families to afford technological equipment updates.^131,136^ The COVID-19 pandemic may, therefore, lead to the deepening of digital inequalities. Implementation experiments are, however, underway in the United Kingdom to try and narrow digital inequalities and ensure suitable access to digital innovations and skills and are discussed below.^137^

A study exploring the views of people (*n* = 65) from five countries with low health literacy to an accessible digital health intervention for type-2 diabetes highlighted that the majority of participants responded positively to most elements of the intervention, despite having low digital literacy.^138^ Thus, highlighting that there may still be a strong inclination to use digital health innovations in those with low digital literacy or access to digital technologies. Increasing the recognition of those most at risk of digital exclusion is key to facilitate the implementation of targeted strategies to improve accessibility and to narrow the skills gap. The independent digital inclusion organisation Citizens Online has used NHS data and produced a map of general practices in England, where patients are not likely to use digital services.^139^ Furthermore, there are now some promising initiatives tackling the issue of digital inclusion including the Leeds Digital Inclusion, 100% Digital Leeds campaign. This is a comprehensive UK approach to support those who would otherwise be digitally excluded within the local area and provides online training, grants to volunteer organisations, a scheme for lending digital devices, help with adopting NHS self-management tools and signposting to reliable health information, as well as auditing of the access to digital tools such as the availability of free Wi-Fi.^140,141^ DevicesDotNow is a UK government-supported initiative for businesses to donate digital devices, dongles, mobile hotspots, and phone sim cards to try to prevent digital exclusion.^142^ The Welsh government has also funded the Wales Co-operative Centre to distribute tablets to those most vulnerable in various healthcare settings including hospitals, hospices, and care homes to facilitate a video consulting service to patients and those at risk of digital exclusion.^143^ Initiatives to bridge the digital divide between nations have also been proposed which could advance digital development in LMIC through cooperation between the United States and various public and private partners.^144^ Issues around accessibility and inclusivity pose real problems. However, targeted initiatives as part of a well thought out implementation strategy could help to tackle this, to facilitate use of digital health innovations in COPD in an inclusive manner. Significant work in this field is required.

### Digital engagement: a key challenge to be overcome

With the rapid advancement of technology and solutions to numerous world problems through digital tools, a key barrier to the successful implementation of digital healthcare solutions will be patient and HCP engagement, rather than the technology itself. Resolving issues discussed above around inclusivity and patients skills will be important, as well as ensuring that apps are acceptable to patients and HCPs without leading to an unacceptable increase in disease-management burden.^65,145^ With this in mind, frameworks have now been described to help the identification and modification of detriments of adherence and engagement.^146,147^ A recent systematic review highlighted numerous barriers for patient engagement, ranging from personal agency and motivation, patients’ skills and values, a lack of understanding, a lack of clinical endorsement and negative digital health experiences or perception of digital innovation quality.^148^ A qualitative study by Slevin *et al.*^65^ reported patients perceiving digital health solutions to offer limited benefits and found them to align poorly with illness or social context. Adherence to digital health app advice has also been shown to be poor, particularly to recommendations on air quality alerts.^149^ Understanding determinants of initiation, persistence and discontinuation of engagement with different parts of multitooled apps will be beneficial to help identify solutions to overcome these issues through clinical trials which incorporate defined engagement endpoints. Determining the presence of patient skills and how these associate with engagement and behaviour changes will also be important to help increase engagement with digital health innovations.

Problems are faced with the ephemeral use of digital technologies and attrition after initial engagement has been widely reported.^50,150^ A survey of health app users found that nearly 75% of people stopped using an app prior to their 10th use.^151^ In line with the law of attrition, this has likely lead to an underestimation of benefit in those who used the app when using an intention-to-treat analysis in clinical trials.^152^ However, an RCT in patients with mild-moderate COPD demonstrated that 65% (*n* = 29) of participants used the app >10 times.^36^ A further RCT reported that 85% (*n* = 21) of patients activated the app, and 40% used the app once a week in line with the 90-day trial minimum recommendation.^40^ However, the level of engagement over longer time periods in different COPD groups is still to be determined. Differences between trials highlight the need to better understand app characteristics and other factors contributing to sustained engagement. Profiling tools and machine-learning approaches that group respiratory patients into clusters based on attitudes, beliefs, and engagement could enable the delivery of personalised interventions or allow provision of interventions for specific self-motivated groups; although this does raise problems of inclusivity discussed above.^153^

#### The importance of user-centred design

User-centred design and personalisation of digital health apps based on patient and HCP needs may be key to improving patient engagement. Yardley *et al.*^154^ described the utility of a ‘person-based’ approach to help incorporate user-specific preferences to aid the successful implementation of and engagement with digital health innovations. Using a mixed methods and in-depth qualitative research approach to tailor digital interventions through an iterative process to meet the needs of the users may help enhance patient engagement.^50^ The RCT discussed above by Farmer *et al.*, which recommended use of the ‘EDGE’ digital health tablet system, was developed following patient workshops with a patient-centred design.^41,42^ This allowed custom algorithms to be developed with personalised alert thresholds and the system was utilised six times a week on average. Furthermore, the study by Lewis *et al.*,^51^ described above, supplemented the quantitative assessment of outcomes following digital provision of PR with qualitative analysis to generate new insights for platform development, barriers to engagement, and the burden to staff.

Alongside development of digital innovations using a user-centred approach, further understanding the relationship between digital health intervention engagement and desired behavioural changes may help to understand how to best meet the needs of patients with COPD and clinicians. Importantly, targeting an improvement in effective engagement with digital health innovations may have a greater impact on changing patient behaviour and outcomes, rather than just increasing the overall engagement, as some types of engagement may not positively benefit desired patient or clinican-orientated outcomes.^50^

The incorporation of behavioural theory, while targeting specific modifiable determinants and understanding how patient factors and skills impact engagement, should also be explored for increasing engagement and adherence, as well as the potential of tokenised rewards following use; although this may have its own drawbacks which have been previously reported.^33,155–157^ Essential research demonstrating the benefit of digital innovations will increase the evidence base for clinicians to point to and could improve both clinician and patient engagement. Services providing counselling to patients about the importance of regular sustained app use may also be needed to support digital health app integration into global health systems.

### Ensuring digital healthcare safety and creating a high-quality evidence base

Ensuring digital healthcare solutions are safe to use in COPD is essential, and various proof-of-concept studies have reported signals of safety and clinical benefit.^36,40,58,158^ However, the use of digital tools is not without risk of adverse outcomes; one RCT reported a higher incidence of adverse musculoskeletal events in those receiving the Internet-mediated pedometer-based exercise intervention.^159^ Other studies reported patients feeling burdened by the responsibility and an increase in self-management tasks, as well as HCPs finding that remote interactions led to less-accurate clinical assessments.^145,160,161^ Other concerns have been reported around the potential of telehealth to lead to overtreatment, as well as promoting the over-dependency of patients on HCPs, particularly in patients with more severe COPD disease.^162–164^ However, the comparative impact of more-complex apps, which provide self-management plans, on healthcare burden is yet to be determined. Some HCPs have reported concerns about telehealth undermining their capacity for holistic surveillance and the difficulty in interpreting the data without the social and physical environmental context.^145,160,161,163,165^ The potential for digital healthcare solutions to decrease direct patient contact time and trust in the healthcare system needs to be explored, as these are known to be essential for patient engagement with self-health behaviours.^166^ The impact of digital healthcare solutions on anxiety around health, addiction to devices and changing of patient behaviours for the worse in response to device feedback also needs to be considered. Training may be important to enable HCPs to adapt to using digital healthcare solutions which will necessitate a new approach to best interact with and manage patients with COPD.^145^ Considerable thought is required as to how best to approach this.

#### Standardised RCTs and high-quality research is now required

Although there are some proof of concept RCTs demonstrating the safety and benefit of different digital health solutions, overall there is a real lack of high-quality evidence in larger RCTs.^167,168^ Due to the pace of digital health development and inevitable inability for research to keep up, the gap in evidence is increasing.^169^ Thus, expanding the evidence base is a priority and will be essential for engagement of healthcare systems, HCPs, and patients with digital health solutions. This will require carefully designed RCTs matched with qualitative studies in various COPD phenotypes, both during stable disease and exacerbations, to also understand clinician and patient concerns and how to overcome these and maximise their engagement.^40,51^ Ensuring the consistency, and cross-trial comparability of evidence will also be important using standardised frameworks which have been proposed.^169–173^

### The problem of scalability and integration into healthcare systems

With the promise of digital health solutions in offering 24/7 support to facilitate self-care to try and meet the unmet COPD need, one of the key issues to be overcome will be the integration into healthcare systems worldwide and engaging all stakeholders and HCPs, as well as providing adequate training. Ensuring interoperability and scalability of apps will be key in facilitating this endeavour, alongside understanding the impact of different technologies on HCP workload and existing services.^174^ Global healthcare systems already have a problem of large amounts of data being produced without frictionless accessibility between services.^175,176^ Poor integration of digital healthcare apps in an already fractured system could just provide another silo of data which may not necessarily be easily interservice accessible. Current digital health approaches in COPD mostly add a digital complexity to already established conventional pathways, rather than reinventing care through adaptive solutions, which may be required. Digital health app output data should be integrated into patients’ records in a frictionless manner to allow clinical decisions, in a similar way to biochemical results.

Progress by consortia such as INTEROPen (www.interopen.org) is being made in improving the standardisation and transfer between devices and healthcare information systems. The successful integration of digital health apps into healthcare systems will require leadership at the global scale, particularly to reach patients and HCPs in LMIC which may lack the infrastructure to support these digital solutions.^39,174,177^ To enable successful implementation and exploitation of digital health technologies, it will be essential to engage and ensure personal commitment of those who direct health organisation leadership and strategy, rather than having the disconnect of relying solely on more junior staff to implement digital health technologies.^178^ Digital maturity assessments (DMAs), a self-assessment mechanism for organisations, have been proposed and could help generate digital roadmaps.^174^ With the use and response to DMAs, national frameworks and infrastructure to allow patient-centred services could be developed. Early development of required digital architecture will be essential to allow effective interaction of information systems, removing barriers for and ensuring engagement of HCP and patients.^179^ Furthermore, it will be important to create an open environment to support emerging care models with necessarily building blocks to enable effective management and sharing of data while ensuring sufficient data protection and information governance, as currently targeted by NHS England.^174^ Innovative solutions, such as blockchain technologies, may have potential in allowing interoperability of systems and digitising trustworthy records independently from single databases, which could be potentially unsafe. This could help to revolutionise current healthcare system logistics and the capacity for information sharing, and aid the implementation of digital technologies into healthcare systems.^157^

### Regulation and providing the right digital interventions is essential

Hundreds of thousands of digital healthcare apps are now available.^180^ However, not all have undergone regulatory approval. Regulation is essential to ensure safety and efficacy but is costly and time-consuming, and there is often a race to market as devices and software can be readily mimicked. This can result in poorer quality products which may be marketed as lifestyle rather than health products to overcome regulation.^181^ Clear regulatory standards may, therefore, need to be tailored to encourage development of high-quality apps which are medically focused.^33^ Close and effective partnership with regulators, companies, and healthcare systems will be required to facilitate appropriate design, regulation, and integration into healthcare systems.^178^ NICE published a digital regulation framework in order to try and bridge the gap of suboptimal evidence around safety and efficacy of digital health innovations. This highlighted the importance of integrating a patient-centred approach into the innovations as well as generating necessary health economics evidence.^182^ A new NHS standard has recently been established based on the Digital Technology Assessment Criteria (DTAC) which will be required to be met for digital technologies for use within the NHS.^183^ NICE are also completing the first assessments of digital technologies using their digital health technologies guidance development standards and decisions on their recommendations are expected soon, alongside publication of an updated framework in 2022.^184,185^

Work by McNamee *et al.*^186^ highlighted the difficulty of accurately delineating the health economic impact of digital health innovations, and the requirement for more-complex economic evaluation methods using a modelling framework, which is capable of accounting for dynamic interactions between patient populations, the environment, the intervention, and healthcare system. The sheer number of apps and lack of a recommendation in most healthcare systems creates confusion and difficulty with both clinician and patient engagement. Building the necessary evidence base to identify those apps which really add value to people’s lives and have potential to improve health will be required, to allow effective regulation and recommendation by regulatory bodies.^178^ Consideration will also be needed around regulation of physiological sensors and the requirement for calibration to ensure the standard of data for clinical and self-management decisions. Sufficient thought will also be needed around: patient consent for data; data security and governance; maintaining confidentiality and anonymity of patient data, both when stored and when in transit; ownership of data; and the potential impact of data on patients’ and their insurance will also be required.^187^

## Limitations of this review and future work

This is a rapidly evolving field and there are an increasing number of small feasibility studies with diverse study and digital health innovation designs which are now being reported. We, therefore, performed a narrative review, aimed at generating new insights and raising questions around the potential of digital healthcare, the potential applications of these evolving innovations, their pitfalls, their barriers to effective utilisation and integration into global healthcare systems, and essential future research. As this was not a formal systematic review, this article may be subject to publication bias, selection bias, and other biases. Furthermore, we do not report the quality of evidence of these small feasibility studies or do a formal comprehensive comparison. However, we do provide the search results and references to allow full appraisal by the reader. Due to the emerging nature of this field, we included abstracts as part of the discussion within this narrative review. However, throughout our discussion, we call for high-quality, standardised RCTs in different COPD populations, as we see generation of an evidence base to be key for overcoming barriers, the ultimate demonstration of safety and efficacy, and to facilitate regulation and integration into healthcare systems. Formal systematic reviews should be performed on emerging evidence in the future.

## Conclusion

COPD remains a debilitating disease and a huge burden to patients and global healthcare systems. Key components of optimal COPD care are the cessation of exposure to pollutants, PR, patient education and self-management, and recognising and management of exacerbations. However, globally patients with COPD face problems of access to healthcare services and the adequate provision of tools and education, particularly in LMIC. Innovative adaptive solutions are required to allow affordable, scalable access to healthcare to empower patients and facilitate effective self-management.

During the COVID-19 pandemic, there has been mass disruption to healthcare services and a potentially long-lasting impact on how COPD services are run. During this period, digital healthcare innovations have generated new interest from both HCPs and patients and are now being rapidly expanded and scaled up. Building the evidence base is now essential to ensure high-quality, standardised, longitudinal research with all relevant endpoints to facilitate high-quality innovations, engagement and essential regulation. The successful incorporation of effective and engaging digital health innovations into healthcare systems to provide digitally augmented care has the potential to remodel global disease management and meet the great unmet clinical need of COPD.

## Supplemental Material

sj-docx-1-tar-10.1177_17534666221075493 – Supplemental material for Digital healthcare in COPD management: a narrative review on the advantages, pitfalls, and need for further research Click here for additional data file. Supplemental material, sj-docx-1-tar-10.1177_17534666221075493 for Digital healthcare in COPD management: a narrative review on the advantages, pitfalls, and need for further research by Alastair Watson and Tom M.A. Wilkinson in Therapeutic Advances in Respiratory Disease
