# Supplementary material for: Digital healthcare in COPD management: a narrative review on the advantages, pitfalls, and need for further research
Source: Ther Adv Respir Dis. 2022 Mar 2;16:17534666221075493. doi: 10.1177/17534666221075493 (PMC8894614; doi:10.1177/17534666221075493)
Supplement: sj-docx-1-tar-10.1177_17534666221075493 – Supplemental material for Digital healthcare in COPD management: a narrative review on the advantages, pitfalls, and need for further research [file sj-docx-1-tar-10.1177_17534666221075493.docx]

Table S1. Search strategies for identifying potential of digital health innovations.

| **Search strategy number** | **Search terms used** | **Number of Results** |
| --- | --- | --- |
| **1** | (("Digital Health" OR “Digital tool*” OR “Digital innovation” OR “digital technolog*” OR “Digital intervention*”) AND ("self-management plan" OR "self management plan" OR "self management*")) AND ("COPD" OR "chronic obstructive pulmonary disease") | 32 |
| **2** | (("Digital Health" OR “Digital tool*” OR “Digital innovation” OR “digital technolog*” OR “Digital intervention*”) AND ("PR" OR "pulmonary rehabilitation") AND ("COPD" OR "chronic obstructive pulmonary disease")) | 14 |
| **3** | (("Digital Health" OR “Digital tool*” OR “Digital innovation” OR “digital technolog*” OR “Digital intervention*”) AND ("inhaler") AND ("COPD" OR "chronic obstructive pulmonary disease")) | 14 |
| **4** | (("Digital Health" OR “Digital tool*” OR “Digital innovation” OR “digital technolog*” OR “Digital intervention*”) AND ("exacerbation*") AND ("COPD" OR "chronic obstructive pulmonary disease")) | 22 |
| **5** | (("Digital Health" OR “Digital tool*” OR “Digital innovation” OR “digital technolog*” OR “Digital intervention*”) AND ("COVID-19" OR "Coronavirus" OR “COVID19” OR “COVID 19”)) | 1,476 |

Formal search strategy performed on PubMed to identify relevant studies which demonstrated the potential of digital health innovations for (**1**) use as self-management plans, for (**2**) providing pulmonary rehabilitation, for (**3**) improving inhaler technique and for (**4**) identifying or early treatment of exacerbations. This strategy was supplemented by an additional search around (**5**) the potential of digital health in COVID-19, as well as a wide general search around digital health in COPD to identify the potential advantages, pitfalls and barriers for utilization. Search results for search strategies 1, 2, 3 and 4 are shown in Tables S2, S3, S4 and S5, respectively. Results for search strategy 5 are no shown. Additional studies, not found through these specific search strategies, were identified from a more general literature search and by reading through reference lists. This narrative review aimed to generate new insights around the potential of digital healthcare, the potential applications of these evolving innovations, their pitfalls, their barriers to effective utilization and integration into global healthcare systems and essential future research. As this was not a systematic review, a full formal search strategy, inclusion criteria or appraisal framework were not used. Relevant studies of interest for inclusion within this narrative review were identified by A.W. and T.W., any conflicts of opinions about which studies should be included were resolved through discussion.

Table S2. Search results for literature around digital health innovations for use as COPD self-management plans

| **Study Number** | **Reference** |
| --- | --- |
| 1 | lwashmi MF, Fitzpatrick B, Davis E, Farrell J, Gamble JM, Hawboldt J. Features of a mobile health intervention to manage chronic obstructive pulmonary disease: a qualitative study. Ther Adv Respir Dis. 2020;14:1753466620951044. |
| 2 | Bashi N, Fatehi F, Mosadeghi-Nik M, Askari MS, Karunanithi M. Digital health interventions for chronic diseases: a scoping review of evaluation frameworks. BMJ Health Care Inform. 2020;27(1). |
| 3 | Bentley CL, Powell L, Potter S, Parker J, Mountain GA, Bartlett YK, et al. The Use of a Smartphone App and an Activity Tracker to Promote Physical Activity in the Management of Chronic Obstructive Pulmonary Disease: Randomized Controlled Feasibility Study. JMIR Mhealth Uhealth. 2020;8(6):e16203. |
| 4 | Boer L, Bischoff E, van der Heijden M, Lucas P, Akkermans R, Vercoulen J, et al. A Smart Mobile Health Tool Versus a Paper Action Plan to Support Self-Management of Chronic Obstructive Pulmonary Disease Exacerbations: Randomized Controlled Trial. JMIR Mhealth Uhealth. 2019;7(10):e14408. |
| 5 | Burkow TM, Vognild LK, Johnsen E, Risberg MJ, Bratvold A, Breivik E, et al. Comprehensive pulmonary rehabilitation in home-based online groups: a mixed method pilot study in COPD. BMC Res Notes. 2015;8:766. |
| 6 | Burkow TM, Vognild LK, Østengen G, Johnsen E, Risberg MJ, Bratvold A, et al. Internet-enabled pulmonary rehabilitation and diabetes education in group settings at home: a preliminary study of patient acceptability. BMC Med Inform Decis Mak. 2013;13:33. |
| 7 | Chan AHY, Pleasants RA, Dhand R, Tilley SL, Schworer SA, Costello RW, et al. Digital Inhalers for Asthma or Chronic Obstructive Pulmonary Disease: A Scientific Perspective. Pulm Ther. 2021;7(2):345-76. |
| 8 | de Batlle J, Massip M, Vargiu E, Nadal N, Fuentes A, Ortega Bravo M, et al. Implementing Mobile Health-Enabled Integrated Care for Complex Chronic Patients: Intervention Effectiveness and Cost-Effectiveness Study. JMIR Mhealth Uhealth. 2021;9(1):e22135. |
| 9. | Ding H, Fatehi F, Maiorana A, Bashi N, Hu W, Edwards I. Digital health for COPD care: the current state of play. J Thorac Dis. 2019;11(Suppl 17):S2210-s20. |
| 10. | Easton K, Potter S, Bec R, Bennion M, Christensen H, Grindell C, et al. A Virtual Agent to Support Individuals Living With Physical and Mental Comorbidities: Co-Design and Acceptability Testing. J Med Internet Res. 2019;21(5):e12996. |
| 11. | Farmer A, Williams V, Velardo C, Shah SA, Yu LM, Rutter H, et al. Self-Management Support Using a Digital Health System Compared With Usual Care for Chronic Obstructive Pulmonary Disease: Randomized Controlled Trial. J Med Internet Res. 2017;19(5):e144. |
| 12. | Houchen-Wolloff L, Orme M, Barradell A, Clinch L, Chaplin E, Gardiner N, et al. Web-Based Self-management Program (SPACE for COPD) for Individuals Hospitalized With an Acute Exacerbation of Chronic Obstructive Pulmonary Disease: Nonrandomized Feasibility Trial of Acceptability. JMIR Mhealth Uhealth. 2021;9(6):e21728. |
| 13. | Janjua S, Banchoff E, Threapleton CJ, Prigmore S, Fletcher J, Disler RT. Digital interventions for the management of chronic obstructive pulmonary disease. Cochrane Database Syst Rev. 2021;4(4):Cd013246. |
| 14. | Joglekar S, Sastry N, Coulson NS, Taylor SJ, Patel A, Duschinsky R, et al. How Online Communities of People With Long-Term Conditions Function and Evolve: Network Analysis of the Structure and Dynamics of the Asthma UK and British Lung Foundation Online Communities. J Med Internet Res. 2018;20(7):e238. |
| 15 | Kjellsdotter A, Andersson S, Berglund M. Together for the Future - Development of a Digital Website to Support Chronic Obstructive Pulmonary Disease Self-Management: A Qualitative Study. J Multidiscip Healthc. 2021;14:757-66. |
| 16 | Knox L, Gemine R, Rees S, Bowen S, Groom P, Taylor D, et al. Using the Technology Acceptance Model to conceptualise experiences of the usability and acceptability of a self-management app (COPD.Pal®) for Chronic Obstructive Pulmonary Disease. Health Technol (Berl). 2020:1-7. |
| 17 | ouri A, Gupta S, Yadollahi A, Ryan CM, Gershon AS, To T, et al. Addressing Reduced Laboratory-Based Pulmonary Function Testing During a Pandemic. Chest. 2020;158(6):2502-10. |
| 18 | North M, Bourne S, Green B, Chauhan AJ, Brown T, Winter J, et al. A randomised controlled feasibility trial of E-health application supported care vs usual care after exacerbation of COPD: the RESCUE trial. NPJ Digit Med. 2020;3:145. |
| 19 | Pinnock H, McKinstry B. Digital technology in respiratory diseases: Promises, (no) panacea and time for a new paradigm. Chron Respir Dis. 2016;13(2):189-91. |
| 20 | Shah SA, Velardo C, Farmer A, Tarassenko L. Exacerbations in Chronic Obstructive Pulmonary Disease: Identification and Prediction Using a Digital Health System. J Med Internet Res. 2017;19(3):e69. |
| 21 | Shaw G, Whelan ME, Armitage LC, Roberts N, Farmer AJ. Are COPD self-management mobile applications effective? A systematic review and meta-analysis. NPJ Prim Care Respir Med. 2020;30(1):11. |
| 22 | Slevin P, Kessie T, Cullen J, Butler MW, Donnelly SC, Caulfield B. A qualitative study of chronic obstructive pulmonary disease patient perceptions of the barriers and facilitators to adopting digital health technology. Digit Health. 2019;5:2055207619871729. |
| 23 | Slevin P, Kessie T, Cullen J, Butler MW, Donnelly SC, Caulfield B. Exploring the potential benefits of digital health technology for the management of COPD: a qualitative study of patient perceptions. ERJ Open Res. 2019;5(2). |
| 24 | Slevin P, Kessie T, Cullen J, Butler MW, Donnelly SC, Caulfield B. A qualitative study of clinician perceptions regarding the potential role for digital health interventions for the management of COPD. Health Informatics J. 2021;27(1):1460458221994888. |
| 25 | Sloots J, Bakker M, van der Palen J, Eijsvogel M, van der Valk P, Linssen G, et al. Adherence to an eHealth Self-Management Intervention for Patients with Both COPD and Heart Failure: Results of a Pilot Study. Int J Chron Obstruct Pulmon Dis. 2021;16:2089-103. |
| 26 | Smith E, Thomas M, Calik-Kutukcu E, Torres-Sánchez I, Granados-Santiago M, Quijano-Campos JC, et al. ERS International Congress 2020 Virtual: highlights from the Allied Respiratory Professionals Assembly. ERJ Open Res. 2021;7(1). |
| 27 | Stellefson M, Chaney B, Chaney D, Paige S, Payne-Purvis C, Tennant B, et al. Engaging community stakeholders to evaluate the design, usability, and acceptability of a chronic obstructive pulmonary disease social media resource center. JMIR Res Protoc. 2015;4(1):e17. |
| 28 | Talboom-Kamp EP, Verdijk NA, Kasteleyn MJ, Harmans LM, Talboom IJ, Numans ME, et al. High Level of Integration in Integrated Disease Management Leads to Higher Usage in the e-Vita Study: Self-Management of Chronic Obstructive Pulmonary Disease With Web-Based Platforms in a Parallel Cohort Design. J Med Internet Res. 2017;19(5):e185. |
| 29 | Taylor A, Lowe DJ, McDowell G, Lua S, Burns S, McGinness P, et al. Remote-Management of COPD: Evaluating the Implementation of Digital Innovation to Enable Routine Care (RECEIVER): the protocol for a feasibility and service adoption observational cohort study. BMJ Open Respir Res. 2021;8(1). |
| 30 | Tighe SA, Ball K, Kensing F, Kayser L, Rawstorn JC, Maddison R. Toward a Digital Platform for the Self-Management of Noncommunicable Disease: Systematic Review of Platform-Like Interventions. J Med Internet Res. 2020;22(10):e16774. |
| 31 | Velardo C, Shah SA, Gibson O, Clifford G, Heneghan C, Rutter H, et al. Digital health system for personalised COPD long-term management. BMC Med Inform Decis Mak. 2017;17(1):19. |
| 32 | Whelan M, Biggs C, Areia C, King E, Lawson B, Newhouse N, et al. Recruiting patients to a digital self-management study whilst in hospital for a chronic obstructive pulmonary disease exacerbation: A feasibility analysis. Digit Health. 2021;7:20552076211020876. |

All search results for search strategy 1, to identify literature around digital health innovations with potential for use as self-management plans. Relevant primary studies of interest were chosen for inclusion in the narrative part of the review. Some review and other article types were read to gain a broader understanding identify of the field and to identify additional relevant studies of interest which are not within this list, but which were also included in the narrative review.

Table 2. Search results for literature around digital health innovations with potential in providing remote pulmonary rehabilitation

| **Study Number** | **Reference** |
| --- | --- |
| 1 | Bentley CL, Powell L, Potter S, Parker J, Mountain GA, Bartlett YK, et al. The Use of a Smartphone App and an Activity Tracker to Promote Physical Activity in the Management of Chronic Obstructive Pulmonary Disease: Randomized Controlled Feasibility Study. JMIR Mhealth Uhealth. 2020;8(6):e16203. |
| 2 | Bourne S, DeVos R, North M, Chauhan A, Green B, Brown T, et al. Online versus face-to-face pulmonary rehabilitation for patients with chronic obstructive pulmonary disease: randomised controlled trial. BMJ Open. 2017;7(7):e014580. |
| 3 | Burkow TM, Vognild LK, Johnsen E, Risberg MJ, Bratvold A, Breivik E, et al. Comprehensive pulmonary rehabilitation in home-based online groups: a mixed method pilot study in COPD. BMC Res Notes. 2015;8:766. |
| 4 | Burkow TM, Vognild LK, Østengen G, Johnsen E, Risberg MJ, Bratvold A, et al. Internet-enabled pulmonary rehabilitation and diabetes education in group settings at home: a preliminary study of patient acceptability. BMC Med Inform Decis Mak. 2013;13:33. |
| 5 | Caille P, Alexandre F, Molinier V, Heraud N. The role of personality traits in inpatient pulmonary rehabilitation response in patients with chronic obstructive pulmonary disease. Respir Med. 2021;190:106680. |
| 6 | Fekete M, Fazekas-Pongor V, Balazs P, Tarantini S, Nemeth AN, Varga JT. Role of new digital technologies and telemedicine in pulmonary rehabilitation : Smart devices in the treatment of chronic respiratory diseases. Wien Klin Wochenschr. 2021;133(21-22):1201-7. |
| 7 | Hognon L, Heraud N, Varray A, Torre K. Adaptive Capacities and Complexity of Heart Rate Variability in Patients With Chronic Obstructive Pulmonary Disease Throughout Pulmonary Rehabilitation. Front Physiol. 2021;12:669722. |
| 8 | Houchen-Wolloff L, Orme M, Barradell A, Clinch L, Chaplin E, Gardiner N, et al. Web-Based Self-management Program (SPACE for COPD) for Individuals Hospitalized With an Acute Exacerbation of Chronic Obstructive Pulmonary Disease: Nonrandomized Feasibility Trial of Acceptability. JMIR Mhealth Uhealth. 2021;9(6):e21728. |
| 9. | Janjua S, Banchoff E, Threapleton CJ, Prigmore S, Fletcher J, Disler RT. Digital interventions for the management of chronic obstructive pulmonary disease. Cochrane Database Syst Rev. 2021;4(4):Cd013246. |
| 10. | Leitl D, Jarosch I, Glöckl R, Schneeberger T, Rembert Koczulla A. [Rehabilitation in pneumology]. Pneumologe (Berl). 2021:1-10. |
| 11. | Maisto M, Diana B, Di Tella S, Matamala-Gomez M, Montana JI, Rossetto F, et al. Digital Interventions for Psychological Comorbidities in Chronic Diseases-A Systematic Review. J Pers Med. 2021;11(1). |
| 12. | Slevin P, Kessie T, Cullen J, Butler MW, Donnelly SC, Caulfield B. Exploring the barriers and facilitators for the use of digital health technologies for the management of COPD: a qualitative study of clinician perceptions. Qjm. 2020;113(3):163-72. |
| 13. | Stellefson M, Alber J, Paige S, Castro D, Singh B. Evaluating Comparative Effectiveness Research Priorities for Care Coordination in Chronic Obstructive Pulmonary Disease: A Community-Based eDelphi Study. JMIR Res Protoc. 2015;4(3):e103. |
| 14. | Zahiri M, Wang C, Gardea M, Nguyen H, Shahbazi M, Sharafkhaneh A, et al. Remote Physical Frailty Monitoring-The Application of Deep Learning-Based Image Processing in Tele-Health. IEEE Access. 2020;8:219391-9. |
| 15 | Zhou H, Park C, Poursina O, Zahiri M, Nguyen H, Torres Ruiz I, et al. Harnessing Digital Health to Objectively Assess Functional Performance in Veterans with Chronic Obstructive Pulmonary Disease. Gerontology. 2021:1-11. |

All search results for search strategy 2, to identify literature around digital health innovations with potential for providing remote pulmonary rehabilitation. Relevant primary studies of interest were chosen for inclusion in the narrative part of the review. Some review and other article types were read to gain a broader understanding identify of the field and to identify additional relevant studies of interest which are not within this list, but which were also included in the narrative review.

Table 3. Search results for literature around digital health innovations with potential for improving inhaler technique

| **Study Number** | **Reference** |
| --- | --- |
| 1 | Biddiscombe MF, Usmani OS. Is there room for further innovation in inhaled therapy for airways disease? Breathe (Sheff). 2018;14(3):216-24. |
| 2 | Blakey JD, Bender BG, Dima AL, Weinman J, Safioti G, Costello RW. Digital technologies and adherence in respiratory diseases: the road ahead. Eur Respir J. 2018;52(5). |
| 3 | Bowler R, Allinder M, Jacobson S, Miller A, Miller B, Tal-Singer R, et al. Real-world use of rescue inhaler sensors, electronic symptom questionnaires and physical activity monitors in COPD. BMJ Open Respir Res. 2019;6(1):e000350. |
| 4 | Cazzola M, Cavalli F, Usmani OS, Rogliani P. Advances in pulmonary drug delivery devices for the treatment of chronic obstructive pulmonary disease. Expert Opin Drug Deliv. 2020;17(5):635-46. |
| 5 | Chan AHY, Pleasants RA, Dhand R, Tilley SL, Schworer SA, Costello RW, et al. Digital Inhalers for Asthma or Chronic Obstructive Pulmonary Disease: A Scientific Perspective. Pulm Ther. 2021;7(2):345-76. |
| 6 | Chen J, Kaye L, Tuffli M, Barrett MA, Jones-Ford S, Shenouda T, et al. Passive Monitoring of Short-Acting Beta-Agonist Use via Digital Platform in Patients With Chronic Obstructive Pulmonary Disease: Quality Improvement Retrospective Analysis. JMIR Form Res. 2019;3(4):e13286. |
| 7 | Dekhuijzen R, Lavorini F, Usmani OS, van Boven JFM. Addressing the Impact and Unmet Needs of Nonadherence in Asthma and Chronic Obstructive Pulmonary Disease: Where Do We Go From Here? J Allergy Clin Immunol Pract. 2018;6(3):785-93. |
| 8 | Kaye L, Gondalia R, Barrett MA, Williams M, Stempel DA. Concurrent Improvement Observed in Patient-Reported Burden and Sensor-Collected Medication Use Among Patients Enrolled in a COPD Digital Health Program. Front Digit Health. 2021;3:624261. |
| 9. | Melani AS. Inhaler technique in asthma and COPD: challenges and unmet knowledge that can contribute to suboptimal use in real life. Expert Rev Clin Pharmacol. 2021;14(8):991-1003. |
| 10. | North M, Bourne S, Green B, Chauhan AJ, Brown T, Winter J, et al. A randomised controlled feasibility trial of E-health application supported care vs usual care after exacerbation of COPD: the RESCUE trial. NPJ Digit Med. 2020;3:145. |
| 11. | O'Dwyer S, Greene G, MacHale E, Cushen B, Sulaiman I, Boland F, et al. Personalized Biofeedback on Inhaler Adherence and Technique by Community Pharmacists: A Cluster Randomized Clinical Trial. J Allergy Clin Immunol Pract. 2020;8(2):635-44. |
| 12. | Sahanic S, Boehm A, Pizzini A, Sonnweber T, Aichner M, Weiss G, et al. Assessing self-medication for obstructive airway disease during COVID-19 using Google Trends. Eur Respir J. 2020;56(5). |
| 13. | Sevinç C, Tertemiz KC, Atik M, Güler N, Ulusoy M, Coşkun F, et al. How were Non-COVID pulmonary patients and diseases affected from COVID-19 pandemic period? Turk Thorac J. 2021;22(2):149-53. |
| 14. | Sloots J, Bakker M, van der Palen J, Eijsvogel M, van der Valk P, Linssen G, et al. Adherence to an eHealth Self-Management Intervention for Patients with Both COPD and Heart Failure: Results of a Pilot Study. Int J Chron Obstruct Pulmon Dis. 2021;16:2089-103. |

All search results for search strategy 3, to identify literature around digital health innovations with potential for improving inhaler technique. Relevant primary studies of interest were chosen for inclusion in the narrative part of the review. Some review and other article types were read to gain a broader understanding identify of the field and to identify additional relevant studies of interest which are not within this list, but which were also included in the narrative review.

Table S4 Search results for literature around digital health innovations with potential for identifying or enabling early treatment of exacerbations

| **Study Number** | **Reference** |
| --- | --- |
| 1 | Blakey JD, Bender BG, Dima AL, Weinman J, Safioti G, Costello RW. Digital technologies and adherence in respiratory diseases: the road ahead. Eur Respir J. 2018;52(5). |
| 2 | Boer L, Bischoff E, van der Heijden M, Lucas P, Akkermans R, Vercoulen J, et al. A Smart Mobile Health Tool Versus a Paper Action Plan to Support Self-Management of Chronic Obstructive Pulmonary Disease Exacerbations: Randomized Controlled Trial. JMIR Mhealth Uhealth. 2019;7(10):e14408. |
| 3 | Bowler R, Allinder M, Jacobson S, Miller A, Miller B, Tal-Singer R, et al. Real-world use of rescue inhaler sensors, electronic symptom questionnaires and physical activity monitors in COPD. BMJ Open Respir Res. 2019;6(1):e000350. |
| 4 | Chan AHY, Pleasants RA, Dhand R, Tilley SL, Schworer SA, Costello RW, et al. Digital Inhalers for Asthma or Chronic Obstructive Pulmonary Disease: A Scientific Perspective. Pulm Ther. 2021;7(2):345-76. |
| 5 | Dekhuijzen R, Lavorini F, Usmani OS, van Boven JFM. Addressing the Impact and Unmet Needs of Nonadherence in Asthma and Chronic Obstructive Pulmonary Disease: Where Do We Go From Here? J Allergy Clin Immunol Pract. 2018;6(3):785-93. |
| 6 | Ding H, Fatehi F, Maiorana A, Bashi N, Hu W, Edwards I. Digital health for COPD care: the current state of play. J Thorac Dis. 2019;11(Suppl 17):S2210-s20. |
| 7 | Easton K, Potter S, Bec R, Bennion M, Christensen H, Grindell C, et al. A Virtual Agent to Support Individuals Living With Physical and Mental Comorbidities: Co-Design and Acceptability Testing. J Med Internet Res. 2019;21(5):e12996. |
| 8 | Fan KG, Mandel J, Agnihotri P, Tai-Seale M. Remote Patient Monitoring Technologies for Predicting Chronic Obstructive Pulmonary Disease Exacerbations: Review and Comparison. JMIR Mhealth Uhealth. 2020;8(5):e16147. |
| 9. | Fekete M, Fazekas-Pongor V, Balazs P, Tarantini S, Nemeth AN, Varga JT. Role of new digital technologies and telemedicine in pulmonary rehabilitation : Smart devices in the treatment of chronic respiratory diseases. Wien Klin Wochenschr. 2021;133(21-22):1201-7. |
| 10. | Gupta P, Wen H, Di Francesco L, Ayazi F. Detection of pathological mechano-acoustic signatures using precision accelerometer contact microphones in patients with pulmonary disorders. Sci Rep. 2021;11(1):13427. |
| 11. | Hall JI, Lozano M, Estrada-Petrocelli L, Birring S, Turner R. The present and future of cough counting tools. J Thorac Dis. 2020;12(9):5207-23. |
| 12. | Houchen-Wolloff L, Orme M, Barradell A, Clinch L, Chaplin E, Gardiner N, et al. Web-Based Self-management Program (SPACE for COPD) for Individuals Hospitalized With an Acute Exacerbation of Chronic Obstructive Pulmonary Disease: Nonrandomized Feasibility Trial of Acceptability. JMIR Mhealth Uhealth. 2021;9(6):e21728. |
| 13. | Janjua S, Banchoff E, Threapleton CJ, Prigmore S, Fletcher J, Disler RT. Digital interventions for the management of chronic obstructive pulmonary disease. Cochrane Database Syst Rev. 2021;4(4):Cd013246. |
| 14. | Karhade AV, Chen AF, Makhni MC, Schwab JH, Simpson AK, Tsai TC. Home Hospital for Orthopaedic Surgery: Opportunities and Challenges of a New Delivery Model. J Bone Joint Surg Am. 2021. |
| 15 | North M, Bourne S, Green B, Chauhan AJ, Brown T, Winter J, et al. A randomised controlled feasibility trial of E-health application supported care vs usual care after exacerbation of COPD: the RESCUE trial. NPJ Digit Med. 2020;3:145. |
| 16 | O'Dwyer S, Greene G, MacHale E, Cushen B, Sulaiman I, Boland F, et al. Personalized Biofeedback on Inhaler Adherence and Technique by Community Pharmacists: A Cluster Randomized Clinical Trial. J Allergy Clin Immunol Pract. 2020;8(2):635-44. |
| 17 | Patalano F, Gutzwiller FS, Shah B, Kumari C, Cook NS. Gathering Structured Patient Insight to Drive the PRO Strategy in COPD: Patient-Centric Drug Development from Theory to Practice. Adv Ther. 2020;37(1):17-26. |
| 18 | Shah SA, Velardo C, Farmer A, Tarassenko L. Exacerbations in Chronic Obstructive Pulmonary Disease: Identification and Prediction Using a Digital Health System. J Med Internet Res. 2017;19(3):e69. |
| 19 | Shaw G, Whelan ME, Armitage LC, Roberts N, Farmer AJ. Are COPD self-management mobile applications effective? A systematic review and meta-analysis. NPJ Prim Care Respir Med. 2020;30(1):11. |
| 20 | Sloots J, Bakker M, van der Palen J, Eijsvogel M, van der Valk P, Linssen G, et al. Adherence to an eHealth Self-Management Intervention for Patients with Both COPD and Heart Failure: Results of a Pilot Study. Int J Chron Obstruct Pulmon Dis. 2021;16:2089-103. |
| 21 | Taylor A, Lowe DJ, McDowell G, Lua S, Burns S, McGinness P, et al. Remote-Management of COPD: Evaluating the Implementation of Digital Innovation to Enable Routine Care (RECEIVER): the protocol for a feasibility and service adoption observational cohort study. BMJ Open Respir Res. 2021;8(1). |
| 22 | Whelan M, Biggs C, Areia C, King E, Lawson B, Newhouse N, et al. Recruiting patients to a digital self-management study whilst in hospital for a chronic obstructive pulmonary disease exacerbation: A feasibility analysis. Digit Health. 2021;7:20552076211020876. |

All search results for search strategy 4, to identify literature around digital health innovations with potential for identifying or enabling early treatment of exacerbations. Relevant primary studies of interest were chosen for inclusion in the narrative part of the review. Some review and other article types were read to gain a broader understanding identify of the field and to identify additional relevant studies of interest which are not within this list, but which were also included in the narrative review.
